# Correction: Suppression of USP18 Potentiates the Anti-HBV Activity of Interferon Alpha in HepG2.2.15 Cells via JAK/STAT Signaling

**DOI:** 10.1371/journal.pone.0159019

**Published:** 2016-07-05

**Authors:** Lin Li, Qing-song Lei, Shu-Jun Zhang, Ling-na Kong, Bo Qin

## Notice of Republication

This article was republished on June 23, 2016, to correct errors introduced while preparing the manuscript for publication. IFN-ɑ appeared incorrectly throughout the article. Please download this article again to view the correct version. The originally published, uncorrected article and the republished, corrected article are provided here for reference.

## Supporting Information

S1 File Originally published, uncorrected article. (PDF)

S2 File Republished, corrected article. (PDF)
