# Supplementary material for: Correction: Suppression of USP18 Potentiates the Anti-HBV Activity of Interferon Alpha in HepG2.2.15 Cells via JAK/STAT Signaling
Source: PLoS One. 2016 Jul 5;11(7):e0159019. doi: 10.1371/journal.pone.0159019 (PMC4933365; doi:10.1371/journal.pone.0159019)
Supplement: S2 File — (PDF) [file pone.0159019.s002.PDF]

PLOS ONE

RESEARCH ARTICLE

Suppression of USP18 Potentiates the Anti-HBV Activity of Interferon Alpha in HepG2.2.15 Cells via JAK/STAT Signaling

Lin Li[1], Qing-song Lei[1], Shu-Jun Zhang[1], Ling-na Kong[1,2], Bo Qin[1]*

1 Department of Infectious Diseases, the First Affiliated Hospital of Chongqing Medical University, Chongqing, 400016, P.R. China, **2** The Nursing College of Chongqing Medical University, Chongqing, 400016, P.R. China

* cqqino@126.com

Abstract

Ubiquitin-specific protease 18 (USP18, also known as UBP43) has both interferon stimulated gene 15 (ISG15) dependent and ISG15-independent functions. By silencing the expression of USP18 in HepG2.2.15 cells, we studied the effect of USP18 on the anti-HBV activity of IFN-α and demonstrated that knockdown of USP18 significantly Inhibited the HBV expression and increased the expression of ISGs. Levels of hepatitis B virus surface antigen (HBsAg), hepatitis B virus e antigen (HBeAg), HBV DNA and intracellular hepatitis B virus core antigen (HBcAg) were dramatically decreased with or without treatment of indicated dose of IFN-α. Suppression of USP18 activated the JAK/STAT signaling pathway as shown by the increased and prolonged expression of phosphorylated signal transducer and activator of transcription 1 (p-STAT1) in combination with enhanced expression of several interferon stimulated genes (ISGs). Our results indicated that USP18 modulates the anti-HBV activity of IFN-α via activation of the JAK/STAT signaling pathway in Hepg2.2.15 cells.

OPEN ACCESS

Citation: Li L, Lei Q-s, Zhang S-J, Kong L-n, Qin B (2016) Suppression of USP18 Potentiates the Anti-HBV Activity of Interferon Alpha in HepG2.2.15 Cells via JAK/STAT Signaling. PLoS ONE 11(5): e0156496. doi:10.1371/journal.pone.0156496

Editor: Haitao Guo, Indiana University, UNITED STATES

Received: January 14, 2016

Accepted: May 16, 2016

Published: May 26, 2016

Copyright: © 2016 Li et al. This is an open access article distributed under the terms of the Creative Commons Attribution License, which permits unrestricted use, distribution, and reproduction in any medium, provided the original author and source are credited.

Data Availability Statement: All relevant data are within the paper and its Supporting Information files.

Funding: This study was supported by grants (81271838) from the National Science Foundation of China, http://www.nsfc.gov.cn. The funder had no role in study design, data collection and analysis, decision to publish, or preparation of the manuscript.

Competing Interests: The authors have declared that no competing interests exist.

Introduction

Chronic hepatitis B (CHB) is a prevalent health problem in the world. Although highly effective vaccines against hepatitis B virus (HBV) are available, HBV infection remains to be one of the most serious health problems and there are still more than 400 million chronic carriers worldwide. Chronic HBV infection can lead to cirrhosis or hepatocellular carcinoma (HCC) and other end-stage liver diseases [1, 2]. Interferon-α is approved as one of the main choices in the treatment for HBV but the sustained virological response in chronic hepatitis B patients is still unsatisfactory. The molecular mechanisms of IFN-α resistance in CHB remain elusive.

Our previous work using Aglient Whole Human Genome Oligo Microarray identified that pre-activation of IFN-α signaling led to differential expression of a subset of interferon stimulated genes (ISGs) and immune-related genes in the pre-treatment liver tissues of treatment responders and non-responders [3]. ISGs were up-regulated more significantly in non-responders compared to responders, which is similar to the findings reported in chronic

hepatitis C infected patients prior to treatment [4, 5]. Among those differentially expressed genes, USP18 and ISG15 are located in the same signal transduction pathway. IFN-α-induced activation of the JAK/STAT signaling pathway lead to the increased expression of several hundred of Interferon stimulated genes (ISGs), which plays an essential role in the anti-viral activity and is closely related to the efficacy of IFN-α treatment [6]. ISG15 is not only an interferon IFN-α/β-inducible ubiquitin-like modifier which can conjugate to cellular substrates to form ISGylated proteins, but also a negative regulator of type I IFN signaling by stabilizing USP18 to prevent IFN-α/β-dependent auto-inflammation [7, 8]. USP18 can specifically cleave the ISG15 from ISG15-conjugated proteins [9, 10]. USP18 was up regulated in the liver of patients treated with peg-IFN-α2b to suppress the JAK/STAT signaling during the first day of the 1-week dosing interval [11]. Previous studies have also shown that USP18-silenced cells are hypersensitive to IFN-α with high levels of ISG15 modified proteins. This phenomenon was also observed in USP18-/- mice, as shown by the more prolonged activation of JAK/STAT signaling with higher levels of ISGylation. USP18 knockout mice show increased antiviral activity aganist a number of viruses including lymphocytic choriomeningitis virus, vesicular stomatitis virus, Sindbis virus and Hepatitis B virus [7, 12–15]. In addition, USP18 can interact directly with proteins involved in immune regulation independent of its ISG15 protease activity [16–18]. Therefore, we hypothesized that USP18 may affect the anti-viral effect of IFN-α on HBV and have predictive value for the efficacy of IFN-α treatment. Furthermore, inhibition of USP18 expression may be an effective way to improve the efficacy of IFN-α treatment.

In this study, we intend to study the effect of silencing USP18 expression by lentivirus-mediated shRNA on the anti-HBV activity of IFN-α in HepG2.2.15 cells, aiming to explore the biological significance of increased expression of USP18 in the pre-treatment liver tissues of treatment non-responders of CHB we previously observed.

Materials and Methods

Cell culture, plasmid transfection and construction of USP18 shRNA lentiviral vectors

Human embryonic kidney cell line HEK-293T (American Type Culture Collection, Manassas, VA) and human hepatoblastoma HepG2 cell line stably transfected by the HBV genome Hepg2.2.15 (preserved in Chongqing Key Laboratory of Infectious diseases and Parasitic diseases) were maintained in high glucose DMEM (Gibco, New York, USA) with 10% fetal bovine serum (Gibco, USA) plus 100μg/ml penicillin and streptomycin and 380μg/ml G418 (sigma-Aldrich, St. Louis, MO, USA) in an incubator at 37°C in 5% CO2 [19]. Stable infected cell lines were selected with 1μg/ml puromycin. HBV replication vector containing 1.1-unit length HBV genome was a gift from Prof. Ni Tang (The Second Affiliated Hospital and the Key Laboratory of Molecular Biology of Infectious Diseases designated by the Chinese Ministry of Education). pcDNA-HBV1.3 (containing 1.3 unit length HBV genome driven by endogenous viral promoter) was preserved in Chongqing Key Laboratory of Infectious diseases and Parasitic diseases. pcDNA3.1-USP18 were from Invitrogen Life Technologies (Invitrogen, USA). Transfection was carried out using Lipofectamine 2000 (Invitrogen, California, USA).

The short hairpin RNA (shRNA) targets of USP18 were designed according to the sequences available in GeneBank (NM_017414). Four pairs of cDNA oligonucleotides targeting the human USP18 mRNA and one negative control shRNA were designed and synthesized. The recombinant vectors were named as shUSP18-1, -2, -3, -4 and shRR, respectively. The recombinant vectors were transformed into E. coli cells and the plasmids were confirmed by DNA sequencing.

HEK 293T cells were seeded at a density of 6×10^5 cells/ml 24 hours prior to transfection at 37°C in 5% CO_2. A total of 45 μg three-plasmid-vector system (20 μg of pGCSIL-GFP plasmids, 15 μg of pHelper 1.0 plasmids and 10 μg of pHelper plasmids 2.0) and 100 μl Lipofectamine 2000 were diluted in 2500μl of MEM medium and then added into the HEK 293T cells. After 48 hours transfection, the medium was harvested and purified through a 0.45 μm filter (Millipore, Billerica, MA) and stored at -80°C. The virus titer was determined by using the hole-by-dilution titer method.

RNA interference assay

Hepg2.2.15 cells were seeded at a density of 8×10^4 cells/ml into 6-well plates 24 hours prior to transduction. An appropriate amount of virus (multiplicity of infection (MOI) = 10) was diluted into the culture medium (containing 5μg/ml of polybrene) on the next day. The recombinant viruses and the control virus were added and the cells were incubated for 6 hours, after which the medium containing virus was removed and replaced with fresh complete medium. Hepg2.2.15 cells were harvested 72 hours after transduction for analyzing USP18 expression. Cells were seeded into 6-well plates for 24 hours and transduced with either shUSP18 or shRR lentivirus, and then were treated with indicated dose of IFN-α (Roche, Basel, Switzerland). After 24-hour-treatment, cells were harvested for analyzing ISGs gene expression.

Quantitative real-time PCR analysis

Total RNA was extracted from Hepg2.2.15 cells using Trizol (Invitrogen, USA) and the cDNAs were synthesized using PrimeScript™ RT Reagent Kit (TaKaRa, DaLian, China) according to the manufacturer's protocol. Quantitative real-time PCR was performed using a Light Cycler system (Bio-Rad Laboratories, Inc., Hercules, CA) using the SYBR Premix Ex Taq™ II Kit (TaKaRa, DaLian, China). The forward and reverse primers are shown in Table 1. Quantification of HBV DNA in the supernatant was performed using quantitative real-time PCR kits (Da-an, Guangzhou, China) according to the instructions.

ELISA

After treatment of indicated dose of IFN-α at various time points, the supernatant was collected for HBsAg and HBeAg analysis. The concentration of HBsAg and HBeAg in the supernatant was detected by ELISA kit (Autobio, ZhengZhou, China) following the manufacturer's protocols.

Western blot analysis

The quantification of protein was performed in HepG2.2.15 cells using Western blot analysis, as previously reported [20]. Rabbit anti-ISG15, rabbit anti-pSTAT1, rabbit anti-STAT1, rabbit anti-pJAK1 and rabbit anti-JAK1 were purchased from cell signaling technology (CST, Danvers, MA), rabbit anti-MxA was purchased from Genetex (Irvine, USA), rabbit anti-IFIT1 was purchased from Bioss (Beijing, China), rabbit anti-USP18 and mouse monoclonal anti-HBcAg were purchased from Abcam (Cambridge, UK), rabbit anti-GAPDH was purchased from Protein Tech Group (Wuhan, China). Secondary antibodies were added for signal detection. An enhanced ECL chemiluminescence detection kit (Millipore, Billerica, MA) was used for detection. The quantification of protein bands related to GAPDH was performed by analysis with FUSION FX imaging system (Vilber, French).

Table 1. Primers for quantitative real-time PCR.

Gene		Sequence 5'-3'
IFIT1	forward	GCAGCCAAGTTTTACCGAAG
	reverse	GCCCTATCTGGTGATGCAGT
ISG15	forward	CGCAGATCACCCAGAAGATT
	reverse	GCCCTTGTTATTCCTCACCA
MxA	forward	GTTTACCAGACTCCGACACGA
	reverse	TTCCAGTGCCTTGATTTGCT
JAK1	forward	TGGGAAATCTGCTACAATGG
	reverse	ATGATGGCTCGGAAGAAAGG
STAT1	forward	TGTTTCATTTGCCACCATCCG
	reverse	ATCCTGAAGATTACGCTTGCT
USP18	forward	CAGACCCTGACAATCCACCT
	reverse	AGCTCATACTGCCCTCCAGA
Beta-actin	forward	GCAAGCAGGAACGATGAG
	reverse	CCATGCCAATGTTGTCTCTT
pgRNA	forward	TCTTGCCTTACTTTTGGAAG
	reverse	AGTTCTTCTTCTAGGGGACC
Total RNA	forward	ACGTCCTTTGTTTACGTCCCGT
	reverse	CCCAACTCCTCCCAGTCCTTAA

doi:10.1371/journal.pone.0156496.t001

Immunofluorescence assay

Cells transduced with shRR or shUSP18 lentiviral vectors were fixed with 4% paraformaldehyde for 20 min, washed three times with cold PBS and then permeabilized in 0.1% Triton X-100 for 15 min. After blocking in 5% goat serum for 1h, cells were incubated with 1:100 anti-STAT1 (CST, Danvers, MA) diluted in blocking solution at 4°C overnight. Cells were washed three times with PBS, and then Cy3 goat anti-rabbit antibody (Abcam, Cambridge, UK) was used as secondary antibody for 1h at room temperature. Nuclei was stained using 5μg/ml 4,6-diamidino-2-phenylindole (DAPI) for 10 min. Cells were washed three times with PBS, and then examined the distribution of STAT1 using fluorescence microscope (Carl Zeiss, Göttingen, Germany).

Statistical analyses

All data are presented as means ± SD. Student's t-test was used for statistical analyses with SPSS 17.0 software (SPSS Inc., Chicago, IL, USA) for Windows. Statistically significant differences were considered when p value <0.05.

Results

USP18 expression is increased in HBV-expressing cells

For better understanding the role of USP18 in HBV infection, we firstly investigated the expression level of USP18 in Hepg2, Hepg2.2.15 and Hepg2 cells transfected with pcDNA-HBV1.3 plasmid or HBV1.1 plasmid. Results showed that the level of USP18 was up regulated in Hepg2.2.15 and Hepg2 cells transfected with HBV1.3 or HBV1.1 expression vectors compared to that of Hepg2 cells (Fig 1). Hepg2.2.15 is a HBV stably transfected cell line constitutively producing HBV. The transient transfection and stable replication of HBV in

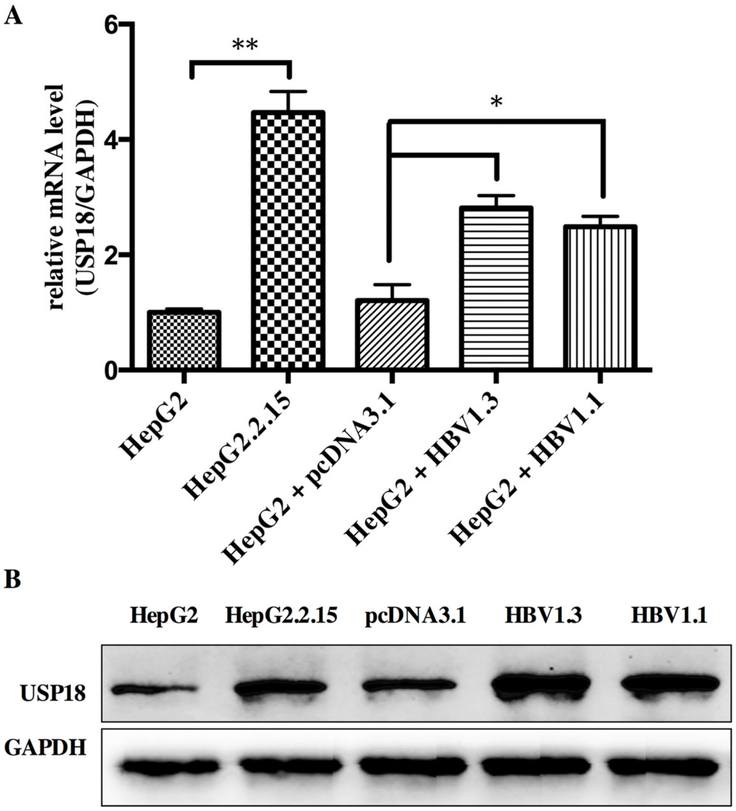

Fig 1. The level of USP18 was up regulated in Hepg2.2.15 and Hepg2 cells transfected with HBV1.3 or HBV1.1 expression vectors compared to that of Hepg2 cells. (A) The mRNA level of USP18 in Hepg2, Hepg2.2.15 and Hepg2 cells transfected with HBV1.3 or HBV1.1 expression vectors. *P < 0.05; **P < 0.01 vs. control. (B) Western blot analysis of USP18 protein level in Hepg2, Hepg2.2.15 and Hepg2 cells transfected with HBV1.3 or HBV1.1 expression vectors.

doi:10.1371/journal.pone.0156496.g001

cells suggest that high expression of USP18 may be associated with the development of chronic HBV infection.

The IFNs were induced to clear exogenous pathogens by the host immune system when viral infection. Type I IFNs bind to the IFNAR to induce STAT1 and STAT2 phosphorylation. The STAT1/2 heterodimers and IFN regulatory factor 9 (IRF9) form a complex and translocate into the nucleus to bind to the interferon stimulate response element (ISRE) in the promoter region of ISGs, resulting in the induction of ISGs against the virus and activation of the adaptive immune response. Reports showed that suppressor of cytokine signaling 1 (SOCS1), the early negative regulator of IFN-α signaling, couldn't inhibit IFN-α-induced phosphorylation and STATs activation without USP18. And for long-lasting refractoriness of IFN-α signaling, USP18 is the key mediator [21]. During acute viral infection, viral replication in secondary lymphatic organs is essential for activating the innate and adaptive immune responses, and the replication is dependent on USP18 [22]. MacParland et al. showed that TNF-α or LPS could target USP18 expression and thus inhibit IFN-signaling [23]. Taken together, we speculate that HBV infection could increase the expression of USP18, which may blunt the IFN-α signaling and thus enhance viral proliferation. Though the underlying mechanism of increased USP18 is currently unclear, this may provide a useful model to further probe the biological roles of USP18 in viral infection.

USP18 expression is efficiently suppressed in lentiviral-vectors-transduced HepG2.2.15 cells

Our previous work using Aglient Whole Human Genome Oligo Microarray have identified that pre-activation of IFN-α signaling led to differential expression of a subset of interferon stimulated genes (ISGs) and immune related genes in the pre-treatment liver tissues of treatment responders and non-responders. In order to study the biological role of increased expression of USP18, we constructed shRNA hairpins against USP18. To determine whether the lentiviral vectors were successfully constructed, the DNA sequencing was performed. The sequencing result (data are not shown) showed that the disturbing sequence was inserted correctly, and the recombinant lentiviral vectors were named as shUSP18-1, -2, -3, -4 and shRR. Different shUSP18 viruses were transduced to examine the effects of USP18 shRNA on HBV replication in HepG2.2.15 cells. Quantitative real-time PCR results indicated that the expression of USP18 mRNA of shUSP18-1 decreased significantly compared to that of shUSP18–2, -3, -4 and the control group (Fig 2A). Therefore, Hepg2.2.15 cells were transduced with shUSP18-1 with various MOI. After 72 hours incubation, the expression of Green Fluorescent Protein (GFP) was observed under the fluorescence microscope. When the MOI was 10, the infection rate was >90%, which was selected as the optimal MOI for the following experiments (Fig 2B). USP18 is an ISG15-specific protease so that decreased USP18 expression can lead to increased ISG15 protein. As expected, USP18 silencing dramatically decreased USP18 protein and increased the free ISG15 protein compared to the control group (Fig 2C). Therefore, shUSP18-1 was selected for further research.

USP18 silencing inhibits HBV expression

To determine whether silencing of USP18 affects HBV expression, we treated Hepg2.2.15 cells at various time points with shUSP18-1 or shRR lentivirus, then the total mRNA and the

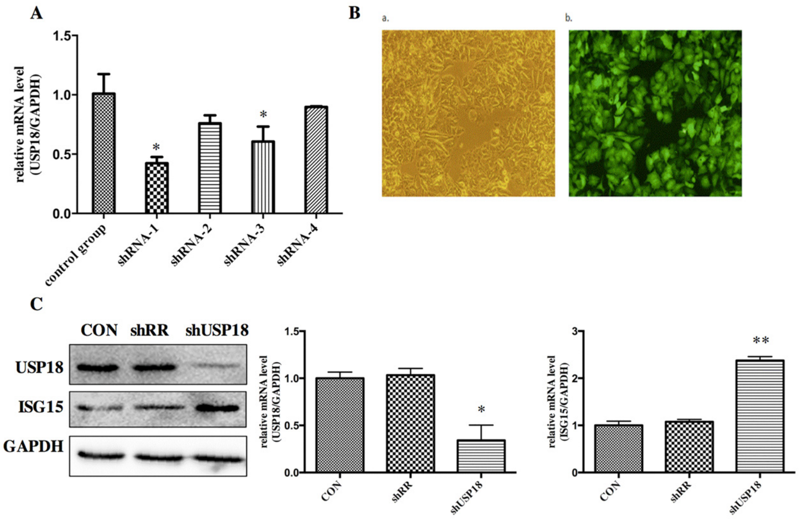

Fig 2. USP18 expression is efficiently suppressed in lentiviral-vectors-transduced HepG2.2.15 cells. (A) Analysis of USP18 mRNA levels. Hepg2.2.15 cells were treated with ShUSP18-1, -2, -3, -4 and shRR. Quantitative real-time PCR analysis was performed to analyze the level of USP18 mRNA 72 hours after transduction. (B) Fluorescence images of Hepg2.2.15 cells transfected for 72 hours in six-well plates with shUSP18–1 per well (MOI = 10). GFP expression was observed under light (a) and fluorescence (b) microscopy (×100). (C) Protein was collected after transduction of shUSP18-1 and levels of USP18 and ISG15 protein in each group were determined by western blot. Lane 1: shUSP18–1 lentivirus group. Lane 2: shRR lentivirus group. Lane 3: normal control group. The results are presented as the means ± SD, n = 3, error bars indicate SD. *P < 0.05; **P < 0.01 vs. control.

doi:10.1371/journal.pone.0156496.g002

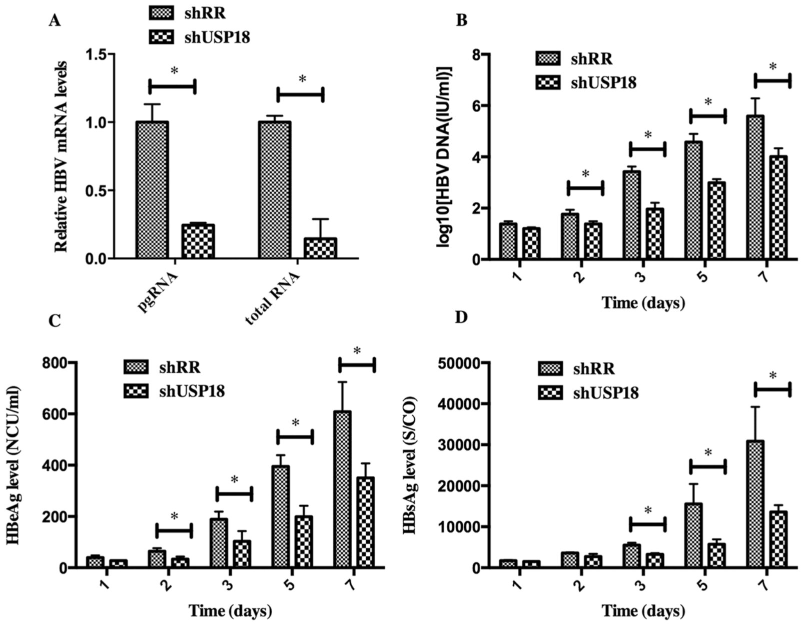

Fig 3. HBV expression was inhibited in Hepg2.2.15 cells after transfected with shUSP18 or shRR lentiviruses. (A) HBV pgRNA and total RNA levels were measured by quantitative real-time PCR. HepG2.2.15 cells were transduced with shUSP18 or shRR lentiviral particles, and then the supernatant was collected at day 1, 2, 3, 5, 7. (B) The extracellular HBV DNA load in the culture was quantified using quantitative real-time PCR kits. The secretion of HBV DNA was suppressed after transduced with shUSP18. (C) and (D) The supernatant was collected for detection of HBsAg and HBeAg using ELISA kits at day 1, 2, 3, 5, 7. The secretion of HBsAg and HBeAg was suppressed after transduced with shUSP18. The results are presented as the means ± SD, n = 3, error bars indicate SD. *P < 0.05; **P < 0.01 vs. control.

doi:10.1371/journal.pone.0156496.g003

supernatant were collected and quantified by quantitative real-time PCR and ELISA according to the manufacturer's protocols. As shown in Fig 3, transduction of shUSP18-1 caused reductions in viral pregenomic RNA (pgRNA) and total RNA levels significantly, indicating that USP18 silencing could affect HBV at the transcriptional level. The secretion of HBV DNA, HBsAg and HBeAg were significantly suppressed compared to the control group, indicating that USP18 silencing could suppress HBV expression efficiently. Notably, the secretion of HBeAg was suppressed more significantly than that of HBsAg.

USP18 silencing enhances the antiviral activity of IFN-α through JAK-STAT signaling pathway. Next, we determined whether USP18 silencing enhances the antiviral activity of IFN-α against HBV. The shUSP18-1-tranduced-HepG2.2.15 cells were treated with indicated dose of IFN-α and cultured for 24 hours, and then qualified the secretion of HBV DNA, HBeAg, HBsAg and intracellular HBcAg. The results suggested that USP18 silencing augments the antiviral effects of IFN-α against HBV expression (Fig 4). We next examined the effect of silencing USP18 on the classical IFN-α-induced JAK-STAT signaling pathway. In cells transduced with shUSP18-1, the mRNA and protein levels of ISGs such as ISG15, MxA and IFIT1 were enhanced in shUSP18-1 lentiviral transduced cells compared to the control group after indicated dose of IFN-α treatment for 24 hours (Fig 5). In line with this, we evaluated the activation status of JAK/STAT signaling. We tested whether USP18 silencing affects the subcellular localization of STAT1 upon stimulation. Immunofluorescence analysis revealed that activation of the JAK/STAT signaling pathway induced the nuclear translocation of STAT1 from the cytoplasm (Fig 6A). In cells transduced with shRR, STAT1 phosphorylation was induced within 1 hour of IFN-α (1000IU/ml) treatment and returned to basal level by 4 hour; In cells

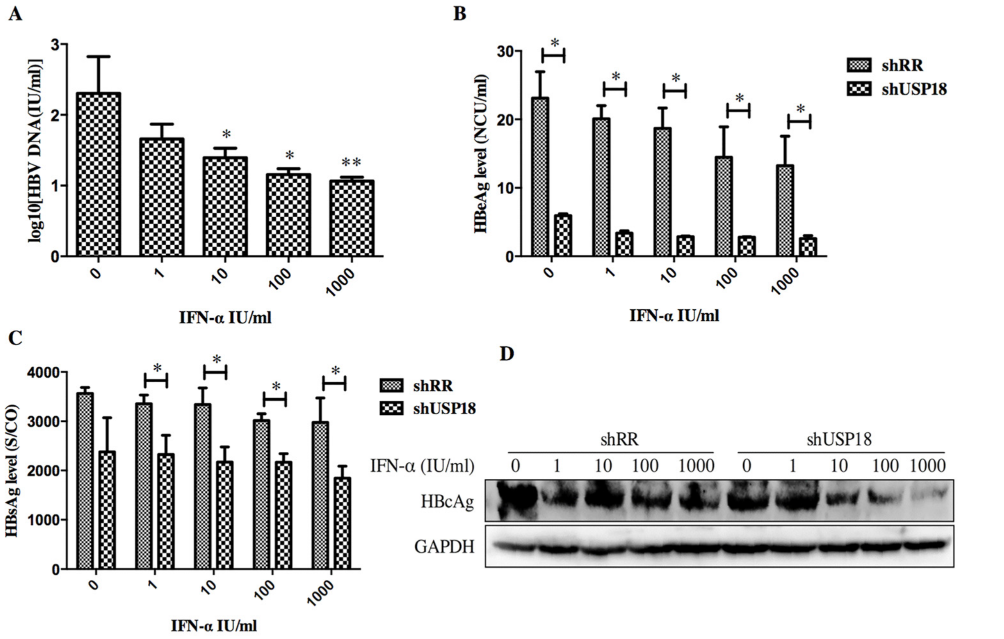

Fig 4. USP18 silencing enhances the antiviral activity of IFN-α against HBV expression in Hepg2.2.15 cells. (A) After treated with indicated dose of IFN-α (0, 1, 10, 100, 1000 IU/ml) in USP18 silenced Hepg2.2.15 cells, the supernatant was collected for detection of HBV DNA load using quantitative real-time PCR kits. (B) and (C) The levels of HBsAg and HBeAg were detected using ELISA kits. The secretion of HBV DNA, HBsAg and HBeAg was significantly inhibited by shUSP18. (D) The level of intracellular HBcAg was quantified by western blot. The results are presented as the means ± SD, n = 3, error bars indicate SD. *P < 0.05; **P < 0.01 vs. control.

doi:10.1371/journal.pone.0156496.g004

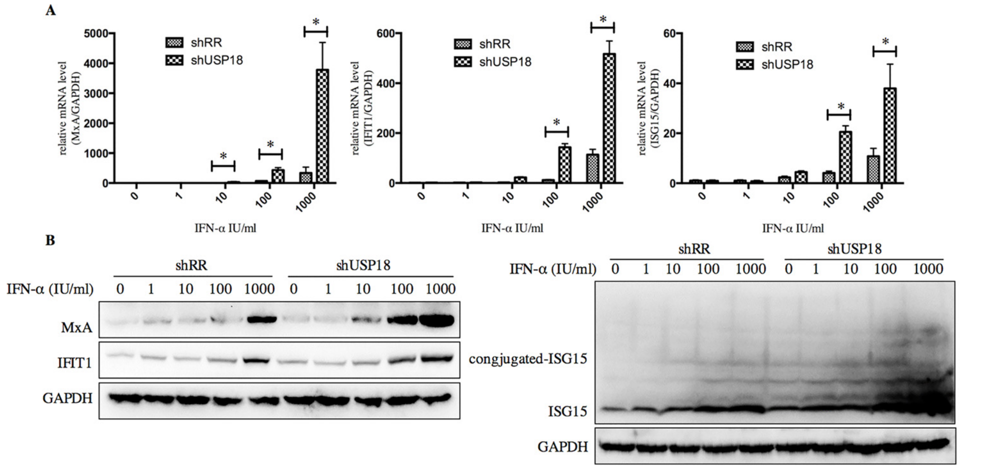

Fig 5. Inhibition of USP18 gene expression in Hepg2.2.15 cells by shUSP18-1 lentivirus increases the antiviral activity of IFN-α. Hepg2.2.15 cells were treated with indicated concentration of IFN-α (0, 1, 10, 100, 1000 IU/ml) for 24 hours after either shUSP18 or shRR lentivirus transduction. (A) and (B) The mRNA and protein levels of ISGs such as ISG15, MxA and IFIT1 were quantified by real-time PCR and western blot separately. The results are presented as the means ± SD, n = 3, error bars indicate SD. *P < 0.05; **P < 0.01 vs. control.

doi:10.1371/journal.pone.0156496.g005

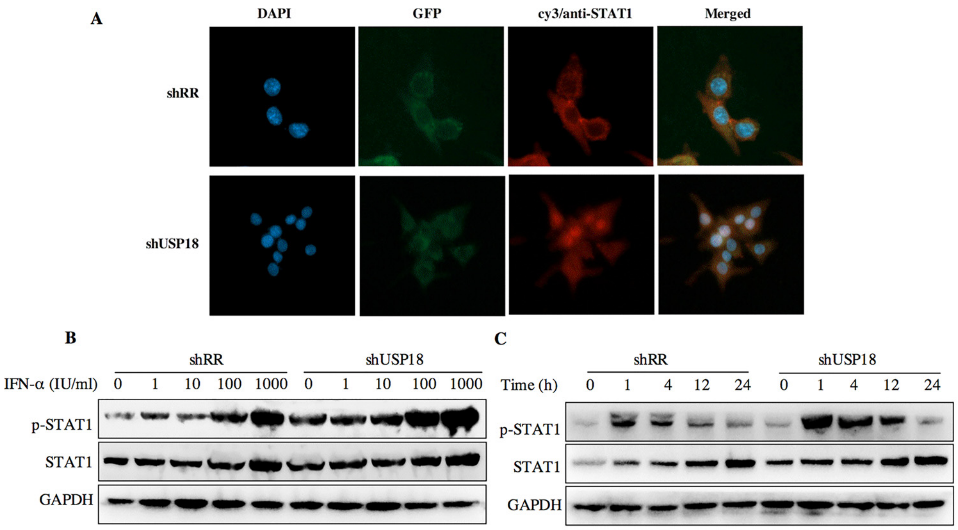

Fig 6. USP18 silencing increases the antiviral activity of IFN-α associated with the JAK-STAT signaling pathway. (A) Hepg2.2.15 cells were treated with shRR or shUSP18 for 48 hours and then subjected to immunofluorescence analysis using a STAT1-specific polyclonal antibody. Blue, DAPI; green, GFP; red, anti-STAT1; original magnification × 400. Hepg2.2.15 cells were treated with indicated concentration of IFN-α (0, 1, 10, 100, 1000 IU/ml) for 20 hours after either shUSP18 or shRR lentivirus transduction. (B) The protein levels of p-STAT1 and STAT1 were quantified by western blot. (C) STAT1 activation was initiated and prolonged in USP18-silenced HepG2.2.15 cells and STAT1 phosphorylation remained 24 hours after treatment compared to the control group.

doi:10.1371/journal.pone.0156496.g006

transduced with shUSP18, STAT1 phosphorylation was prolonged and remained 24 hours after same dose of IFN-α treatment (Fig 6C). These results collectively suggested that silencing USP18 activated IFN-α signaling, JAK/STAT signaling, in a prolonged fashion. In addition, IFN-α stimulation can cause a state of refractoriness in which a second stimulus can no longer induce IFN signaling. Lack of USP18 was shown to abrogate this desensitizing effect of IFN-α in vivo [24], whereas USP18 expression was sufficient to establish this state of refractoriness [25]. To monitor IFN-mediated desensitization, Hepg2.2.15 cells were treated with IFN-α, which induced STAT1 phosphorylation after 1h. Subsequently, cells received a second IFN-α treatment 8h later and p-STAT1 was analyzed 1h after the second treatment. The initial treatment of IFN-α induced p-STAT1 both in the shRR and shUSP18 group, whereas the second treatment of IFN-α induced stronger phosphorylation of STAT1 in the shUSP18 group compared to the control group (S1 Fig). This desensitizing effect indicates that lack of USP18 in the Hepg2.2.15 cells could abrogate this desensitizing effect of IFN-α and enhance the antiviral activity of IFN-α.

To further confirm the role of USP18 in HBV replication, we transfected with pcDNA3.1-USP18 in USP18 silenced cells. After transfection, the intracellular pgRNA and total RNA levels and the secretion of HBV particles were up regulated and the over-expression of ISGs was rescued (S2 Fig), which indicated that the HBV replication was regulated via USP18 expression. Taken together, we concluded that USP18 potentiates the anti-HBV activity of IFN-α by targeting the JAK/STAT signaling pathway in Hepg2.2.15 cells.

Discussion

CHB is a prevalent and severe health problem, which can result in cirrhosis or HCC and other end-stage liver diseases. Although IFN-α is widely used in clinical therapy for hepatitis B and

hepatitis C, only 30%-40% CHB patients can realize HBeAg seroconversion and 50%-60% patients with genotype 1 cannot realize the sustained virological response (SVR). The molecular mechanisms of IFN-α resistance in CHB still remain incompletely understood. It is necessary to develop effective ways to solve those problems and have a better understanding of the mechanisms of IFN-α resistance.

Based on our previous microarray study in the pre-treatment liver tissues of IFN-α treatment responders and non-responders, we found that a series of ISGs were up regulated more significantly in the non-responders group [3]. Two of those genes are USP18 and ISG15 which are closely correlated and in the same ISG15/USP18 signaling. Various studies indicated that activation of ISG15/USP18 pathway is involved in treatment non-response in both HCV and HBV patients [20]. USP18 is an interferon-stimulated gene that can specifically cleave the ISG15 protein from its conjugated proteins. Over expression of ISG15 stimulates HCV replication and enhances the antiviral activity of IFN-α [4]. Interestingly, ISG15-/- and Ube1L-/- mice have similar sensitivity to IFN-α to the parental USP18-/- mice, indicating that USP18 regulates the sensitivity to IFN-α in an ISG15-independent manner. Furthermore, several studies reported that USP18 played a crucial role in the long-term desensitization to IFN in mouse and can boost the anti-proliferation effects of the IFNs [26, 27]. Therefore, USP18 is regarded as a potent negative regulator of IFN-α signaling. Indeed, USP18 binds to IFNAR2 to inhibit type I IFN signaling and subsequently disrupting IFNAR2-JAK1 interaction, thus negatively regulating IFN signaling cascades [10]. In addition, overexpression of USP18 is involved in the proliferation, specific immune response and apoptosis of several kinds of tumors [21, 28–31]. These data indicated that inhibiting the expression of USP18 could be a promising strategy to improve the efficacy of IFN-α treatment. HepG2.2.15 cell is a sub-line derived from human hepatoblastoma HepG2 cell line, which stably transfected with the HBV genome and can secrete HBV DNA, HBsAg and HBeAg. It is widely used to explore the virus-host interactions of chronic hepatitis B infection in vitro.

Firstly, we investigated the expression level of USP18 in Hepg2, Hepg2.2.15 and Hepg2 cells transiently transfected with HBV1.3 plasmid or HBV1.1 plasmid. Results showed that USP18 expression was up regulated in Hepg2.2.15 cells and Hepg2 cells transfected with HBV1.3 or HBV1.1 expression plasmids compared to that of Hepg2 cells (Fig 1), which indicated that USP18 might play a role in the HBV replication process.

For further understanding the role of USP18 in IFN-α signaling, we used specific targeted lentiviral vectors to inhibit the expression of USP18 in Hepg2.2.15 cells. We constructed four recombinant lentiviral vectors specifically targeting USP18 gene, and then quantitative real-time PCR, observation of GFP protein and western blot were carried out to identify the most effective target for further experiment (Fig 2). Results indicated that shUSP18-1 lentiviral vector could suppress the expression of USP18 significantly. Then, we analyzed the intracellular pgRNA and total RNA levels and secretion of HBV DNA, HBsAg and HBeAg in Hepg2.2.15 cells after transduction of either shUSP18-1 or shRR lentiviral vectors. Results demonstrated that the levels of pgRNA and total RNA were decreased significantly. The secretion of HBsAg and HBeAg in Hepg2.2.15 cells were down regulated significantly by transfection of shUSP18-1 (Fig 3), indicating that USP18 silencing could suppress HBV at differential levels.

Next, we treated the shUSP18-transducted cells with different dose of IFN-α (0, 1, 10, 100, 1000 IU/ml) for analyzing the level of mRNA and protein by quantitative real-time PCR and western blot separately. We evaluated the effect of shUS18-1 and IFN-α on the expression of ISGs, which can enhance the antiviral activity of IFN-α. The mRNA expression of ISG15, MxA, and IFIT1 were augmented in shUSP18-silenced group with IFN-α treatment compared to the control group (Fig 5). We therefore evaluated the activation status of JAK/STAT

signaling, a classical IFN-α induced signaling, by looking at the subcellular localization and phosphorylation level of STAT1. Immunofluorescence analysis revealed that activation of the JAK/STAT signaling pathway induced the nuclear translocation of STAT1 from the cytoplasm (Fig 6A). Specifically, IFN-α induced the phosphorylation of STAT1 and USP18 silencing enhanced the STAT1 activation and phosphorylation for a longer periods of time compared to the control group (Fig 6C), leading to increased expression of ISGs, and thus enhanced the antiviral activity against HBV in Hepg2.2.15 cells. Meanwhile, we analyzed the expression level of intracellular HBcAg and collected the supernatant for analyzing HBV DNA, HBsAg and HBeAg. Results showed that expression the level of intracellular HBcAg and secretion of HBV DNA, HBsAg and HBeAg were suppressed significantly after IFN-α treatment in shUSP18-transducted HepG2.2.15 cells (Fig 4). Murray et al. had showed that knockdown of USP18 increases alpha 2a interferon signaling and induction of ISGs but does not increase antiviral activity in Huh7 cells, while the study of Randall et al. reported a more potent effect [5, 15]. It is possible that the differentially expressed host genes at differential concentration of shUSP18 could contribute to altered consequences of IFN signaling. In the present study, we observed the increasing of IFN signaling as well as antiviral activity. We hypothesized that the shRNA lentiviral vectors were transduced into HepG2.2.15 cells to interpret the expression of USP18 and the stable clones were selected with puromycin, which could induce a differential profile of gene expression and lead to a differential immune status, and thus showed a potent antiviral activity. In addition, lacking USP18 can abrogated the refractory status induced by IFN-α stimulation, which indicates that lack of USP18 in the Hepg2.2.15 cells could enhance the antiviral activity of IFN-α (S1 Fig).

Notably, the inhibitory effect of USP18 silencing on the secretion of HBeAg was more significant than that of HBsAg. HBeAg is associated with cirrhosis and HCC. Early seroconversion of HBeAg is a common goal in clinical treatment and could benefit all CHB patients [32]. In the present study, the secretion of HBeAg was decreased early (2d) than that of HBsAg (3d) after USP18 silencing. And after treatment of IFN-α, the secretion of HBsAg in the shUSP18-transduced cells was decreased at a much higher concentration of IFN-α (1 IU/ml) than that of HBeAg (0 IU/ml). This is consistent with the clinical processes of IFN-α treatment. We supposed that USP18 silencing could promote the expression of ISGs and dozens of immune-related genes (data are not shown), which could help inhibiting HBV expression and secretion of HBV serological markers efficiently.

Conclusions

In conclusion, we have successfully constructed and selected the most effective recombinant lentiviral vector for USP18 shRNA expression in Hepg2.2.15 cells for the first time, which significantly increase the antiviral activity of IFN-α and its influence is associated with the IFN-α signaling pathway, JAK-STAT, indicating that USP18 can be used as a candidate target to explore chronic hepatitis B progression and gene therapy.

Supporting Information

S1 Fig. Lack of USP18 in the Hepg2.2.15 cells abrogates the desensitizing effect of IFN-α. Hepg2.2.15 cells were treated with IFN-α and p-STAT1 was analyzed by western blot 1h after the treatment. Subsequently, cells received a second IFN-α treatment 8h later and p-STAT1 was analyzed 1h after the second treatment.
(TIFF)

S2 Fig. Overexpression of USP18 rescues anti-HBV effect by USAP18 silencing. (A) HepG2.2.15 cells were transfected with pcDNA3.1-USP18 or control plasmid. The level of USP18, pgRNA and total RNA was determined by quantitative real-time PCR 72 hours after transfection. (B) HepG2.2.15 cells were transfected with pcDNA3.1-USP18 or control plasmid, and then the supernatant was collected at day 1, 2, 3, 4, 5. The extracellular HBV DNA, HBeAg, HBsAg in the culture was quantified using quantitative real-time PCR kits or ELISA kits. (C) USP18-silenced cells were transfected with pcDNA3.1-USP18 or control plasmid, the level of ISGs proteins were determined by western blot. The results are presented as the means ± SD, n = 3, error bars indicate SD. $^{*}P < 0.05$ vs. control.
(TIFF)

Author Contributions

Conceived and designed the experiments: BQ. Performed the experiments: LL QL LK SZ. Analyzed the data: LL BQ. Wrote the paper: LL BQ.

References

1. Lee WM. 1997. Hepatitis B virus infection. N Engl J Med 337(24): 1733–1745. PMID: 9392700
2. European Association for the Study of the Liver. 2012. EASL clinical practice guidelines: Management of chronic hepatitis B virus infection. J Hepatol. 57(1): 167–185. doi: 10.1016/j.jhep.2012.02.010 PMID: 22436845
3. Xiao C, Qin Bo, Chen L, Liu H, Zhu Y, Lu X. 2012. Preactivation of the interferon signaling in liver is correlated with non-response to interferon alpha therapy in patients chronically infected with hepatitis B virus. Journal of Viral Hepatitis 19(2): e1–e10. doi: 10.1111/j.1365-2893.2011.01471.x PMID: 22239505
4. Chen L, Sun J, Meng L, Heathcote J, Edwards AM, McGilvray ID. 2010. ISG15, a ubiquitin-like interferon-stimulated gene, promotes hepatitis C virus production in vitro: implications for chronic infection and response to treatment. Journal of General Virology 91(Pt 2), 382–388. doi: 10.1099/vir.0.015388-0 PMID: 19846672
5. Randall G, Chen L, Panis M, Fischer AK, Lindenbach BD, Sun J, et al. 2006. Silencing of USP18 Potentiates the Antiviral Activity of Interferon Against Hepatitis C Virus Infection. Gastroenterology 131(5): 1584–1591. PMID: 17101330
6. Giannakopoulos NV, Luo JK, Papov V, Zou W, Lenschow DJ, Jacobs BS, et al. 2005. Proteomic identification of proteins conjugated to ISG15 in mouse and human cells. BiochemBiophys Res Commun 336(2): 496–506.
7. Kim JH, Luo JK, Zhang DE. 2008. The level of hepatitis B virus replication is not affected by protein ISG15 modification but is reduced by inhibition of UBP43 (USP18) expression. J Immunol 181(9): 6467–6472. PMID: 18941237
8. Zhang X, Bogunovic D, Payelle-Brogard B, Francois-Newton V, Speer SD, Yuan C, et al. 2015. Human intracellular ISG15 prevents interferon-α/β over-amplification and auto-inflammation. Nature 517 (7532): 89–93. doi: 10.1038/nature13801 PMID: 25307056
9. Malakhov MP, Malakhova OA, Kim KI, Ritchie KJ, Zhang DE. 2002. UBP43 (USP18) specifically removes ISG15 from conjugated proteins. J Biol Chem 277(12): 9976–9981. PMID: 11788588
10. Malakhova OA, Kim KI, Luo JK, Zou W, Kumar KG, Fuchs SY, et al. 2006. UBP43 is a novel regulator of interferon signaling independent of its ISG15 isopeptidase activity. EMBO J 25(11): 2358–2367. PMID: 16710296
11. Dill MT, Makowska Z, Trincucci G, Gruber AJ, Vogt JE, Filipowicz M, et al. 2014. Pegylated IFN-α regulates hepatic gene expression through transient Jak/STAT activation. J Clin Invest 124(4):1568–1581. doi: 10.1172/JCI70408 PMID: 24569457
12. Burkart C, Arimoto K, Tang T. 2013. USP18 deficient mammary epithelial cells create an antitumour environment driven by hypersensitivity to IFN-λ and elevated secretion of Cxcl10. EMBO Mol Med 5 (7): 967–982. doi: 10.1002/emmm.201201864 PMID: 23681607
13. Colonne PM, Sahni A, Sahni SK. 2011. Rickettsia conorii infection stimulates the expression of ISG15 and ISG15 protease UBP43 in human microvascular endothelial cells. Biochem Biophys Res Commun 416(1–2): 153–158. doi: 10.1016/j.bbrc.2011.11.015 PMID: 22100648

14. Kim KI, Malakhova OA, Hoebe K, Yan M, Beutler B, Zhang DE. 2005. Enhanced antibacterial potential in UBP43-deficient mice against Salmonella typhimurium infection by up-regulating type I IFN signaling. J Immunol 175(2): 847–854. PMID: 16002682

15. Murray EJ, Burden F, Horscroft N, Smith-Burchnell C, Westby M. 2011. Knockdown of USP18 increases α-2a interferon signaling and induction of interferon-stimulating genes but does not increase antiviral activity in Huh7 cells. Antimicrob Agents Chemother 55(9): 4311–4319. doi: 10.1128/AAC.00644-11 PMID: 21709085

16. Liu X, Li H, Zhong B, Blonska M, Gorjestani S, Yan M, et al. 2013. USP18 inhibits NF-κB and NFAT activation during Th17 differentiation by deubiquitinating the TAK1-TAB1 complex. J Exp Med 210(8): 1575–1590. doi: 10.1084/jem.20122327 PMID: 23825189

17. Malhotra S, Morcillo-Suárez C, Nurtdinov R, Rio J, Sarro E, Moreno M, et al. 2013. Roles of the ubiquitin peptidase USP18 in multiple sclerosis and the response to interferon-β treatment. Eur J Neurol 20(10): 1390–1397. doi: 10.1111/ene.12193 PMID: 23700969

18. Salajegheh M, Kong SW, Pinkus JL, Walsh RJ, Liao A, Nazareno R, et al. 2010. Interferon-Stimulated Gene 15 (ISG15) Conjugates Proteins in Dermatomyositis Muscle with Perifascicular Atrophy. Ann Neurol 67(1): 53–63. doi: 10.1002/ana.21805 PMID: 20186858

19. Sells MA, Chen ML, Acs G. 1987. Production of hepatitis B virus particles in Hep G2 cells transfected with cloned hepatitis B virus DNA. Proc Natl Acad Sci U S A. 84(4):1005–9. PMID: 3029758

20. Li Y, Li S, Duan X, Liu B, Yang C, Zeng P, et al. 2014. Activation of endogenous type I IFN signaling contributes to persistent HCV infection. Rev Med Virol 24(5): 332–342. doi: 10.1002/rmv.1795 PMID: 24806972

21. Sarasin-Filipowicz M, Wang X, Yan M, Duong FH, Poli V, Hilton DJ, et al. 2009. Alpha Interferon Induces Long-Lasting Refractoriness of JAK-STAT Signaling in the Mouse Liver through Induction of USP18/UBP43. Mol Cell Biol 29(17): 4841–4851. doi: 10.1128/MCB.00224-09 PMID: 19564419

22. Honke N, Shaabani N, Zhang DE, Iliakis G, Xu HC, Häussinger D, et al. 2013. Usp18 Driven Enforced Viral Replication in Dendritic Cells Contributes to Break of Immunological Tolerance in Autoimmune Diabetes. PloS Pathog 9(10): e1003650. doi: 10.1371/journal.ppat.1003650 PMID: 24204252

23. MacParland SA, Ma XZ, Chen L, Khattar R, Cherepanov V, Selzner M, et al. 2016. LPS and TNF-α inhibit interferon-signaling in hepatocytes by increasing USP18 expression. Journal of Virology 02557–15.

24. Ketscher L, Hannß R, Morales DJ, Basters A, Guerra S, Goldmann T, et al. 2015. Selective inactivation of USP18 isopeptidase activity in vivo enhances ISG15 conjugation and viral resistance. PNAS 112 (5): 1577–82. doi: 10.1073/pnas.1412881112 PMID: 25605921

25. François-Newton V, Magno de Freitas Almeida G, Payelle-Brogard B, Monneron D, Pichard-Garcia L, Piehler J, et al. 2011. USP18-based negative feedback control is induced by type I and type III interferons and specifically inactivates interferon α response. PLoS ONE 6(7): e22200. doi: 10.1371/journal.pone.0022200 PMID: 21779393

26. Potu H, Sqorbissa A, Brancolini C. 2010. Identification of USP18 as an important regulator of the susceptibility to IFN-alpha and drug-induced apoptosis. Cancer Res 70(2): 655–665. doi: 10.1158/0008-5472.CAN-09-1942 PMID: 20068173

27. Burkart C, Fan JB, Zhang DE. 2012. Two independent mechanisms promote expression of an N-terminal truncated USP18 isoform with higher DeISGylation activity in the nucleus. J Biol Chem 287(7): 4883–4893. doi: 10.1074/jbc.M111.255570 PMID: 22170061

28. Duex JE, Comeau L, Sorkin A, Purow B, Kefas B. 2011. USP18 Regulates Epidermal Growth Factor (EGF) Receptor Expression and Cancer Cell Survival via MicroRNA-7. J Biol Chem 286(28): 25377–25386. doi: 10.1074/jbc.M111.222760 PMID: 21592959

29. Guo Y, Chinyengetere F, Dolinko AV, Lopez-Aguiar A, Lu Y, Galimberti F, et al. 2012. Evidence for the ubiquitin protease UBP43 as an antineoplastic target. Mol Cancer Ther 11(9): 1968–1977. doi: 10.1158/1535-7163.MCT-12-0248 PMID: 22752428

30. Makki MS, Rusteshouser EC, Huff V. 2013. Ubiquitin specific protease 18 (USP18) is a WT1 transcriptional target. Exp Cell Res 319(5): 612–622. doi: 10.1016/j.yexcr.2012.12.021 PMID: 23291318

31. Hong B, Li H, Lu Y, Zhang M, Zheng Y, Qian J, et al. 2014. USP18 is crucial for IFN-γ-mediated inhibition of B16 melanoma tumorigenesis and antitumor immunity. Mol Cancer 13:132. doi: 10.1186/1476-4598-13-132 PMID: 24884733

32. Pang YJ, Tan XJ, Li DM, Zheng ZH, Lei RX, Peng XM. 2013. Therapeutic potential of furin inhibitors for the chronic infection of hepatitis B virus. Liver Int 33: 1230–1238. doi: 10.1111/liv.12185 PMID: 23617302
